# Effect of opt-in versus opt-out framing on trial recruitment: a study within a trial of the GAMEPAD randomized trial

**DOI:** 10.1016/j.ahj.2025.107285

**Published:** 2025-10-03

**Authors:** Tayyab Shah, Samantha Coratti, David Farraday, Laurie Norton, Charles Rareshide, Jingsan Zhu, Michael G. Levin, Sae-Hwan Park, Scott M. Damrauer, Jay S. Giri, Neel P. Chokshi, Benjamin M. Jackson, Mitesh S. Patel, Alexander C. Fanaroff

**Affiliations:** a Department of Medicine, Perelman School of Medicine, University of Pennsylvania, Philadelphia, PA,; b Penn Center for Health Incentives and Behavioral Economics, University of Pennsylvania, Philadelphia, PA,; c Department of Medical Ethics and Health Policy, University of Pennsylvania, Philadelphia, PA,; d Corporal Michael J. Crescenz VA Medical Center, Philadelphia, PA,; e Penn Cardiovascular Outcomes, Quality, and Evaluative Research Center, University of Pennsylvania, Philadelphia, PA,; f Penn Cardiovascular Institute, University of Pennsylvania, Philadelphia, PA,; g Division of Vascular Surgery, University of Pennsylvania, Philadelphia, PA,; h Leonard Davis Institute of Health Economics, University of Pennsylvania, Philadelphia, PA,; i Penn Center for Digital Cardiology, University of Pennsylvania, Philadelphia, PA,; j Division of Vascular Surgery, Lehigh Valley Heart and Vascular Institute, Allentown, PA,; k Ascension Health, St. Louis, MO

**Keywords:** Study within a trial, recruitment, embedded randomized controlled trial, exercise, peripheral artery disease, behavioral economics, gamification, health behavior

## Abstract

Directly contacting eligible participants with an offer to join a randomized clinical trial (RCT) is an efficient recruitment method, but the effect of different outreach strategies on enrollment fraction and completion of the trial protocol is uncertain. In a study within a trial (SWAT) of an RCT testing a physical activity intervention in patients with peripheral artery disease, eligible patients were randomized to receive an email with an invitation to join the study and a link to the trial’s online platform (“opt-in”) or to receive an email framing participation as part of the standard of care followed by telephone outreach from a study coordinator (“opt-out”). Among 5176 participants contacted by unsolicited email (3909 opt-in, 1267 opt-out), enrollment fraction was 1.0% in the opt-in arm (*n* = 39) versus 3.6% in the opt-out arm (*n* = 45) (OR 3.65, 95% CI 2.37–5.64); there were no significant differences between opt-in and opt-out participants in the rate of completion of trial protocol steps. This SWAT of recruitment strategies demonstrates the potential for opt-out framing and active outreach to increase enrollment fraction without compromising protocol completion in direct-to-participant RCTs.

In the United States, low participation rates in clinical trials prolong study timelines and increase costs.^1^ In addition, select populations (younger patients, males, White patients, college educated, higher socioeconomic status) are often overrepresented in clinical trials, potentially limiting their external validity and limiting access to historically under-represented populations.^2,3^ Directly contacting participants electronically with a passive offer to join a clinical trial is an efficient recruitment method, but may further bias enrollment away from marginalized populations.^4^ Various strategies have been shown to improve recruitment, including open-label versus blinded clinical trials and active telephone outreach to potential subjects, although their effects are modest at best and further evidence based strategies are needed.^5^ Another potential strategy comes from behavioral economics research which has shown that framing participation in programs like retirement savings and organ donation as the default (opt-out) rather than framing nonparticipation as the default (opt-in) increases participation while maintaining informed choice.^6^ In the clinical setting, opt-out framing increased participation in clinical programs like colon cancer screening and home-based monitoring after myocardial infarction;^7,8^ however, the effect of opt-in versus opt-out framing on trial recruitment and enrollment of marginalized populations remains uncertain and requires further study.^5^ As such, we conducted a randomized recruitment study within a trial (SWAT) of the Gamification-Augmented hoMe-based Exercise for Peripheral Artery Disease (GAMEPAD) randomized controlled trial, a trial testing an intervention to increase physical activity in patients with peripheral arterial disease (PAD).^9,10^ In this SWAT, potential participants were randomly assigned to be contacted for participation in GAMEPAD using traditional opt-in framing versus opt-out framing and active outreach.

## Methods

The detailed methods and primary results of the GAMEPAD trial (NCT04536012) have been published previously.^9,10^ The opt-in/opt-out SWAT presented here was prespecified and separately registered at clinicaltrials.gov (NCT04536038); the protocol is available in Supplementary Methods 1. The parent trial and SWAT were approved by the University of Pennsylvania Institutional Review Board. Trial Forge Guidance 4 was followed in reporting the results of this SWAT.^11^

Briefly, the parent trial enrolled adult patients with PAD from a single health system. Eligible patients were primarily identified through the health system’s clinical data warehouse, but some were also direct referrals from cardiologists or vascular surgeons. Eligible patients (who were not direct referrals) were contacted by unsolicited email and offered participation in the study. Amenable participants were directed to the study website (Way to Health) where they created an account, provided informed consent, and completed baseline survey assessments. Qualifying participants were mailed a wrist-worn wearable device that could track step counts (Fitbit Inspire 1 and 2). After activation, patients entered a 2-week run-in period where their baseline step count was assessed. Those with too high (> 7,500/day) and too low (< 1000/day) step counts were excluded and the remaining set numerical goals for increasing their step counts. These patients were then randomized 1:1 to attention control or to behaviorally-designed gamification with 24-week follow-up of their step counts (16-week intervention period and 8-week follow-up period). The primary outcome was change in daily steps from baseline through the intervention period. In a recruitment SWAT, eligible patients who were not directly referred to the study by a clinician were randomized, originally in a 7:1 ratio and later in a 3:1 ratio to maximize enrollment fraction and meet enrollment targets among a limited population of eligible patients, to receive 1 of 2 different unsolicited emails at time of first contact—1 that framed participation in the traditional opt-in manner and another framing participation in an opt-out manner (Supplementary Methods 2). The randomization sequence was developed by a statistician otherwise unaffiliated with the project who sent a list of patient names (from a master list of patients planned to receive outreach) to study coordinators weekly with each patient’s randomization assignment. Treatment assignment was therefore masked to study coordinators until after randomization. The study coordinators then contacted the patients by email with messaging consistent with their randomized group. Due to the nature of the study, neither research coordinators nor participants were blinded to treatment assignment, but investigators, including the study statistician, were blinded until analyses were completed. Patients randomized to the opt-in framing received 1 email that described the GAMEPAD study and instructed them to visit the study website to enroll in the study, or to contact the study coordinator with questions or for assistance in enrolling. There was no follow-up for opt-in patients who did not respond. Patients randomized to the “opt-out” framing received an email that framed participation in the study as part of the standard of care and were informed that a study coordinator would call them in the coming week to enroll them in the study unless they opted out of participation. All patients in the opt-out arm then received a telephone call follow-up from a study coordinator to invite enrollment. Although the email was framed in an “opt-out” manner, it was not truly an opt-out strategy because participants still were required to provide affirmative informed consent before being enrolled. The study sent enrollment outreach emails from October 20, 2020 to May 9, 2023 and the ratio for opt-in vs opt-out framing was changed to 3:1 on November 17, 2020.

The primary outcome was the enrollment fraction (number of patients that enrolled divided by total contacted) which is reported by initial outreach strategy (opt-in versus opt-out) overall and in key subgroups. We also present patient attrition through study steps, final enrollment demographics, and completion of key study steps by opt-in/opt-out status. Numerical variables are presented as means and standard deviations with comparisons made by the student t-test while categorical variables are presented as numbers and percents with comparisons made using the Chi-squared test or Fisher exact test, as appropriate. Odds Ratios (OR) for patient attrition through study steps and enrollment fractions by subgroup were calculated using contingency tables, with 95% confidence intervals (CI) calculated using the log-transformed OR and its standard error. Logistic regression models were used to determine the interaction between race and sex and the effect of opt-in versus opt-out status on enrollment fraction. We used linear mixed-effect regression models to determine the interaction between opt-in versus opt-out status and the effect of the intervention on change from baseline in daily steps. There was no formal sample size calculation conducted for this SWAT, and sample size was determined by the number of patients requiring outreach to meet enrollment goals in the parent trial. SAS version 9.4 (SAS Institute, Inc., Cary, NC) was used for all analyses.

## Results

In total, 5176 potentially eligible participants were contacted by unsolicited email; 3909 randomized to opt-in and 1267 randomized to opt-out. Ultimately, 84 of these participants were enrolled in the study and randomized to gamification or attention control; an additional 19 participants were directly referred to the study by clinicians and were not included in the opt-in versus opt-out SWAT. Baseline characteristics of patients randomized to opt-in and opt-out framing were similar (Supplementary Table).

With regards to the primary endpoint, 39 participants randomized to opt-in framing and 45 participants randomized to opt-out framing were enrolled in GAMEPAD (enrollment fraction 1% vs 3.6%; OR 3.65, 95% CI 2.37–5.64; *P* <.001) (Figure). Among Black patients, enrollment fraction was 0.3% in the opt-in arm versus 3.1% in the opt-out arm (OR 9.78; 95% CI 2.63–36.38; *P* <.001); among women, enrollment fraction was 1.0% in the opt-in arm versus 4.0% in the opt-out arm (OR 4.11; 95% CI 2.13–7.94; *P* <.001). There were no significant interactions between the effect of opt-in versus opt-out framing and participant sex (interaction *P* =.21) or race (interaction *P* =.55). In addition, compared with opt-in patients, opt-out patients were more likely to navigate to the Way to Health website and create an account (10.7 vs 4.4%; OR 2.60, 95% CI 2.06–3.28; *P* <.001) (Table 1), and, among those who created an account, more likely to provide informed consent (81.6 vs 69.9%; OR 1.91, 95% CI 1.11–3.28; *P* =.02). Among patients who consented to the study, opt-out patients were also significantly more likely to complete all baseline surveys (74.8% vs 62.0%; OR 1.81, 95% CI 1.03–3.20, *P* =.04) but there were no significant differences in the proportion that completed other steps.

Among participants ultimately enrolled in GAMEPAD, there were no significant differences by opt-in or opt-out status in the proportion who submitted step data for > 60% of days during the intervention period or completed the end of intervention or end of follow-up questionnaires. Participants randomized to opt-out submitted step count data on a greater proportion of participant-days (92.3 vs 85.8%, *P* <.001). There were no significant differences in baseline characteristics between the opt-in and opt-out patients (Table 2). However, in the opt-out group there was a numerically higher proportion of females (47% vs 41%), Black patients (20% vs 8%), patients without college degrees (53% vs 36%), and patients with household incomes < $50,000 (38% vs 21%). There was no significant interaction between opt-in versus opt-out status and the effect of the intervention on change from baseline in daily steps (interaction *P* =.38 for the intervention period and 0.55 for the follow-up period).

## Discussion and conclusion

This randomized recruitment SWAT of the GAMEPAD trial demonstrates that using an opt-out framework with active outreach in clinical trial recruitment increased enrollment fraction over threefold compared with an opt-out, passive outreach strategy. Opt-out patients were more than twice as likely to start the study enrollment process and more likely to complete the enrollment process. Importantly, after enrollment, opt-out patients followed the trial protocol and improved step counts at similar rates to opt-in patients, demonstrating that the opt-out framework did not result in enrollment of relatively less interested or motivated patients. Moreover, the largest gains in enrollment fraction were seen among Black patients. In addition, while the differences in baseline characteristics between patients enrolled by the opt-in and opt-out strategies were not statistically significant in this small trial, there were substantially more participants that were Black, female, and had markers of low socioeconomic status enrolled from the opt-out arm than the opt-in arm.

This study has several limitations. We are unable to quantify the cost of each recruitment strategy since we did not prospectively collect relevant information such as the time required to make patient calls. We are also unable to isolate the effect of opt-out framing from active telephone outreach, both of which have been shown to increase enrollment fraction.^5^ Similarly, the opt-out strategy employed in this trial was not truly “opt-out” as patients still needed to provide affirmative informed consent to be enrolled. Thus, while we do not demonstrate the full effect of a complete opt-out strategy, such a strategy is only feasible in a small subset of clinical trials that pose minimal risk in which a waiver of informed consent can be obtained.^12,13^ Still, this SWAT of recruitment strategies adds to the existing body of literature^5,7,8^ demonstrating that opt-out framing and active outreach can increase enrollment fraction and also demonstrates its potential to enhance representativeness.

## Supplementary Material

1

**Supplementary materials** Supplementary material associated with this article can be found, in the online version, at doi:10.1016/j.ahj.2025.107285.

## Figures and Tables

**Figure.**
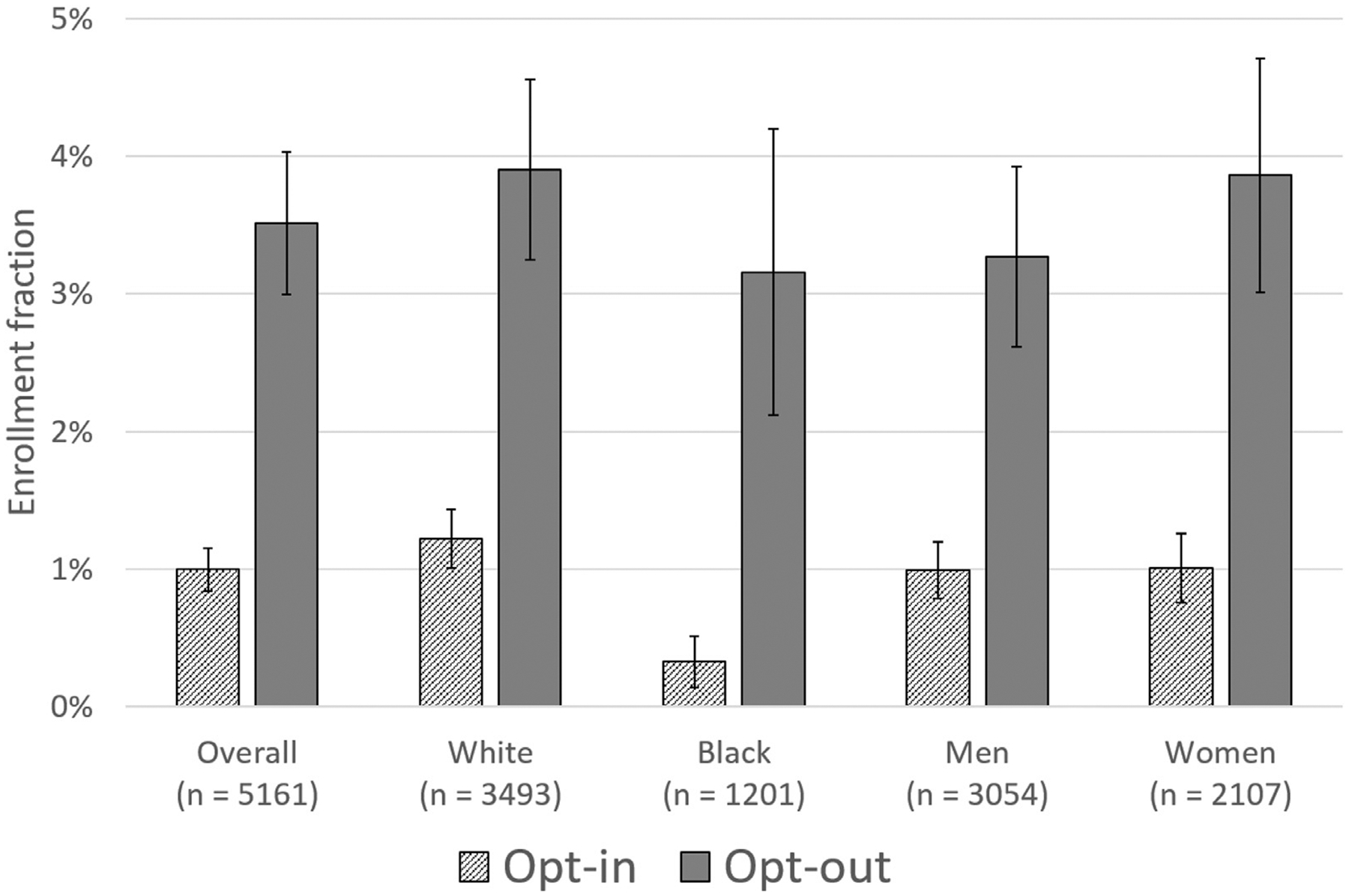
Enrollment fraction by opt-in versus opt-out approach overall and by subgroup. Enrollment fraction (the number of participants who enrolled in the study divided by the number approached) was > 3-fold higher with the opt-out approach, with consistent results across subgroups. Error bars represent standard error of the mean.

**Table 1.** Participant flow in opt-in and opt-out arms of the study

	Opt-in	Opt-out	Odds ratio (95% CI)	*P* value
Contacted	3,909	1,267		
Created account in way to health	173 (4.4%)	136 (10.7%)	2.60 (2.06–3.28)	< .001
Consented	121 (69.9%)	111 (81.6%)	1.91 (1.11–3.28)	.02
Completed all baseline surveys	75 (62.0%)	83 (74.8%)	1.82 (1.03–3.20)	.04
Activated fitbit	59 (78.7%)	71 (85.5%)	1.60 (0.70–3.66)	.26
Completed baseline period and eligible to be randomized	39 (66.1%)	45 (63.4%)	0.89 (0.43–1.83)	.75
Submitted step data for > 60% of days through 16 weeks	33 (84.6%)	42 (93.3%)	2.55 (0.59–10.95)	.21
Completed 16-week survey	36 (92.3%)	39 (86.7%)	0.54 (0.13–2.33)	.41
Completed 24-week survey	36 (92.3%)	37 (82.2%)	0.39 (0.10–1.57)	.18

Percentages in each row are based on the total number of patients that completed the step above. The number of patients who completed baseline monitoring and were randomized is used as the total number for all percentages regarding follow-up completion.

**Table 2.** Baseline characteristics of patients enrolled in GAMEPAD via opt-in and opt-out pathways

Baseline characteristics	Overall (*n* = 84)	Opt-in (*n* = 39)	Opt-out (*n* = 45)	*P* value
Age, y	69.8 (8.7)	70.2 (10.7)	69.4 (6.5)	.84
Male	47 (56%)	23 (59%)	24 (53.3%)	.69
Race				.26
White	67 (79.8%)	32 (82.1%)	35 (77.8%)	
Black	12 (14.3%)	3 (7.7%)	9 (20%)	
Asian	2 (2.4%)	2 (5.1%)	0 (0%)	
Hispanic	0 (0%)	0 (0%)	0 (0%)	
Other	3 (3.6%)	2 (5.1%)	1 (2.2%)	
Education				.27
Some high school	2 (2.4%)	0 (0%)	2 (4.4%)	
High school graduate	12 (14.3%)	4 (10.3%)	8 (17.8%)	
Some college	24 (28.6%)	10 (25.6%)	14 (31.1%)	
College graduate	46 (54.8%)	25 (64.1%)	21 (46.7%)	
Annual household income				.22
<$50,000	25 (29.8%)	8 (20.5%)	17 (37.8%)	
$50,000 to $100,000	28 (33.3%)	15 (38.5%)	13 (28.9%)	
>$100,000	31 (36.9%)	16 (41%)	15 (33.3%)	
BMI, kg/m^2^	30.3 (6.5)	30.1 (6.2)	30.5 (6.9)	.53
Current smoking	11 (13.1%)	5 (12.8%)	6 (13.3%)	.94
Hypertension	69 (82.1%)	34 (87.2%)	35 (77.8%)	.20
Hyperlipidemia	59 (70.2%)	24 (61.5%)	35 (77.8%)	.26
Diabetes	30 (35.7%)	11 (28.2%)	19 (42.2%)	.33
Prior MI	23 (27.4%)	8 (20.5%)	15 (33.3%)	.19
Stroke	9 (10.7%)	3 (7.7%)	6 (13.3%)	.41
Heart failure	10 (11.9%)	2 (5.1%)	8 (17.8%)	.17
COPD	14 (16.7%)	5 (12.8%)	9 (20%)	.59
Kidney disease	14 (16.7%)	7 (17.9%)	7 (15.6%)	.10
Baseline daily step count	4260 (1786)	4463 (1880)	4084 (1702)	.34
Walking impairment questionnaire				
Distance	44.3 (34.7)	44.8 (32.9)	44 (36.5)	.92
Speed	37.5 (25.3)	37.5 (23.7)	37.6 (26.9)	.99
Stairs	43.4 (29)	43.3 (28.6)	43.4 (29.7)	.98
Overall	41.7 (26.2)	41.8 (25.6)	41.7 (26.9)	.97
San diego claudication questionnaire				.72
Classic claudication	18 (21.4%)	7 (17.9%)	11 (24.4%)	
Atypical leg pain	44 (52.4%)	22 (56.4%)	22 (48.9%)	
No symptoms	22 (26.2%)	10 (25.6%)	12 (26.7%)	

Numerical variables are presented as mean (standard deviation) while categorical variables are presented as numbers (percents).
