# Supplementary material for: Effect of opt-in versus opt-out framing on trial recruitment: a study within a trial of the GAMEPAD randomized trial
Source: Am Heart J. Author manuscript; Available in PMC 2026 Apr 17. (PMC13088173; doi:10.1016/j.ahj.2025.107285)
Supplement: 1 [file NIHMS2162667-supplement-1.docx]

**SUPPLEMENTAL MATERIAL**

**Supplemental Methods 1:** Protocol for the GAMEPAD opt-in versus opt-out recruitment SWAT

**Supplemental Methods 2:** Email template for opt-in and opt-out patients

**Supplemental Table:** Characteristics of potential participants randomized to opt-in and opt-out framing

**Supplemental Methods 1:** Study protocol

**Opt-in versus opt-out clinical trial enrollment**

**among patients with peripheral artery disease:**

**A GAME PAD substudy**

Version 1

February 28, 2020

1. **Background**

Research studies are hampered by low participation rates, threatening these studies’ external validity, prolonging study timelines, and increasing costs.(1,2) Behavioral economics research has shown that framing retirement savings and organ donation participation as opt-out rather than opt-in increases participation in these programs while maintaining informed choice.(3) In healthcare, an opt-out approach has also been shown to increase influenza vaccination and human immunodeficiency virus screening.(4,5) More recently, a randomized controlled trial testing opt-in versus opt-out approaches to colorectal cancer screening by fecal occult blood testing showed that the opt-out approach increased participation rates nearly 3-fold.(6)

In clinical trials of home-based monitoring for patients with myocardial infarction and a behavioral intervention for patients with poorly-controlled diabetes mellitus, sub-studies have shown that an opt-out approach increased enrollment compared with an opt-in approach.(7,8) In a trial of a home-based monitoring program for patients with myocardial infarction, eligible patients were enrolled using a traditional opt-in approach.(7) These patients were sent a recruitment letter after their myocardial infarction, contacted via phone and consented for enrollment, and, after consent, sent a remote monitoring medication bottles, entered into a system configured with financial incentives, reminders, and social support. A separate cohort of patients, made ineligible for participation by their insurance type, was sent configured medication bottles along with their recruitment letters to create an opt-out frame. In the opt-in arm, 16% of patients agreed to participate in the study, compared with 38% in the opt-out arm. In a trial of a behavioral intervention for patients with poorly controlled diabetes mellitus, patients were randomized to receive a letter informing them that they had been enrolled in a clinical trial (opt-out) versus a standard recruitment letter (opt-in).(8) In the opt-in arm, 13% of patients agreed to participate compared with 38% in the opt-out arm.

Though opt-out trial participation has increased enrollment in two prior studies, it is uncertain whether it will do the same in a clinical trial intended to promote physical activity in patients with peripheral artery disease (PAD), as these patients differ from those included in the myocardial infarction and diabetes trial in several ways, and the intervention differs as well. We therefore seek to conduct a randomized substudy of the proposed GAME PAD randomized controlled trial, evaluating the effect of opt-out versus opt-in framing of clinical trial enrollment.

1. **Study Objectives**

The objectives of this study are the following:

1. To determine the effect of an opt-out versus an opt-in approach to enrollment in a clinical trial enrolling patients with peripheral artery disease on the proportion of patients contacted enrolled
2. To determine the effect of an opt-out versus an opt-in approach to enrollment in a clinical trial enrolling patients with peripheral artery disease on the clinical characteristics of patients who decide to enroll in the study
3. **Sub-Study Design and Procedures**

**3.1 General overview**

The GAME PAD study is a two-arm randomized controlled trial aimed at evaluating whether a home-based walking program with automated coaching augmented with gamification and behavioral economic principles improves functional capacity in patients with PAD. The study will randomize 100 patients with PAD to a 16-week home-based walking program using goal-setting alone (attention control) or to the same home-based walking program with automated coaching augmented with gamification and behavioral economic principles. The study will be conducted using the Way to Health platform, and patients will enroll and consent for participation online, with available help from a study coordinator by telephone. This sub-study will test whether opt-in or opt-out framing of study participation upon initial contact affects the proportion of patients who ultimately enroll in the study and the demographic characteristics of enrolled patients.

**3.2. Site**

This GAME PAD substudy will enroll patients from the University of Pennsylvania Health System, which consists of five large hospitals including the Hospital of the University of Pennsylvania, Penn Presbyterian Medical Center, Pennsylvania Hospital, and Chester County Hospital.

**3.3. Patient Selection Criteria**

Patients are eligible to be included in the study if they are ≥ 18 years of age, have been seen in the University of Pennsylvania Health System, and have an ICD-10 code consistent with peripheral artery disease (I70.2x, I70.3x-I70.7x, I73.9) or peripheral artery disease on their problem list. There are no exclusion criteria.

Please refer to the separate protocol for inclusion and exclusion criteria for the main GAME PAD study.

**3.4. Substudy procedures**

**3.4.1 Randomization**

Eligible patients will be contacted via email to determine their interest in participation in the GAME PAD study. Patients will be randomized 7:1 to an email message framing trial participation in a standard opt-in manner versus an email message framing trial participation in an opt-out manner.

**3.4.2 Study arms**

Patients randomized to opt-in framing will be instructed to visit the Way to Health website to enroll in the study, or to call or email the study coordinator with questions or for assistance in enrolling. Patients randomized to opt-out framing will receive an email that frames participation in the study as part of the standard of care, and will be informed that a study coordinator will be calling them in the coming days to start enrollment in the study unless they opt out of participation.

**3.4.3 Data collection**

Baseline characteristics of patients randomized to each framing method will be abstracted from the electronic health record and Penn Data Store, and will include age, sex, race/ethnicity, and medical comorbidities. Whether contacted patients created an account on the Way to Health website and/or ultimately consented for enrollment in the study will be captured.

**3.4.4 Outcomes**

For all outcomes, we will compare patients initially presented with study participation via opt-in framing versus opt-out framing. The primary outcome will be the proportion of patients that enroll in the study. Exploratory outcomes, among patients that ultimately enrolled in the study, will include the proportion of enrolled patients that failed to complete the study, change in daily step count from baseline to the end of the GAME PAD intervention and the end of follow-up (overall and stratified by overall GAME PAD study arm – gamification intervention versus attention control), and change in patient-reported symptom scores from baseline to the end of the GAME PAD intervention and the end of follow-up (overall and stratified by overall GAME PAD study arm)

**3.4.5 Informed consent**

Because the trial tests different methods of approaching patients prior to enrollment, informed consent cannot feasibly be obtained and we will request a waiver of informed consent

1. **Statistical Analysis**

We will compare the primary outcome and attrition rate between patients initially presented with study participation via opt-in framing versus opt-out framing using the chi-square method. Change in step count from baseline through the intervention period and follow-up will be compared using paired t tests, as will change in patient reported symptom scores from baseline through the intervention period and the end of follow up. The primary outcome will be analyzed using the intention-to-treat principle, grouping patients by framing of study participation at initial contact. Other outcomes, among patients that ultimately enrolled in the GAME PAD study, will similarly group patients by framing of study participation at initial contact.

# **5. Human Research Protection**

**5.1 Data confidentiality**

Paper-based records will be kept in a secure location and only be accessible to personnel involved in the study. Computer-based files will only be made available to personnel involved in the study through the use of access privileges and passwords. Wherever feasible, identifiers will be removed from study-related information. Precautions are in place to ensure the data are secure by using passwords and encryption, because the research involves web-based surveys.

**5.2 Subject confidentiality**

Research material will be obtained from the electronic health record. The data to be collected include age, sex, race/ethnicity, and medical comorbidities. Research material that is obtained will be used for research purposes only. The study identification number, and not other identifying information, will be used on all data collection instruments. All study staff will be reminded to appreciate the confidential nature of the data collected and contained in these databases. The Penn Medicine Academic Computing Services (PMACS) will be the hub for the hardware and database infrastructure that will support the project and is where the Way to Health web portal is based. The PMACS is a joint effort of the University of Pennsylvania's Abramson Cancer Center, the Cardiovascular Institute, the Department of Pathology, and the Leonard Davis Institute. The PMACS provides a secure computing environment for a large volume of highly sensitive data, including clinical, genetic, socioeconomic, and financial information. Among the IT projects currently managed by PMACS are: (1) the capture and organization of complex, longitudinal clinical data via web and clinical applications portals from cancer patients enrolled in clinical trials; (2) the integration of genetic array databases and clinical data obtained from patients with cardiovascular disease; (3) computational biology and cytometry database management and analyses; (4) economic and health policy research using Medicare claims from over 40 million Medicare beneficiaries. PMACS requires all users of data or applications on PMACS servers to complete a PMACS-hosted cybersecurity awareness course annually, which stresses federal data security policies under data use agreements with the university. The curriculum includes Health Insurance Portability and Accountability Act (HIPAA) training and covers secure data transfer, passwords, computer security habits and knowledge of what constitutes misuse or inappropriate use of the server. We will implement multiple, redundant protective measures to guarantee the privacy and security of the participant data. All investigators and research staff with direct access to the identifiable data will be required to undergo annual responsible conduct of research, cybersecurity, and HIPAA certification in accordance with University of Pennsylvania 334 regulations. Data will be stored, managed, and analyzed on a secure, encrypted server behind the University of Pennsylvania Health System (UPHS) firewall. This server was created for projects conducted by the Penn Medicine Nudge Unit related to physician and patient behavior at UPHS. All study personnel that will use this data are listed on the IRB application and have completed training in HIPAA standards and the CITI human subjects research. Data access will be password protected. Whenever possible, data will be deidentified for analysis.

**5.3 Subject privacy**

For the purposes of this sub-study, participants will not engage in any study-related procedures where privacy could be violatd.

**5.4 Data disclosure**

The following entities, besides the members of the research team, may receive protected health information (PHI) for this research study: The Office of Human Research Protections at the University of Pennsylvania -Federal and state agencies (for example, the Department of Health and Human Services, the National Institutes of Health, and/or the Office 364 for Human Research Protections), or other domestic or foreign government bodies if required by 365 law and/or necessary for oversight purposes.

**5.5 Data safety and monitoring**

The Principal Investigator will be responsible for monitoring the study. For the purposes of this sub-study, participants will not engage in any study-related procedures, and risk will be minimal.

**5.6 Risk/benefit**

**5.6.1 Potential study risks**

The only risk posed to participants is breach of confidentiality. We will minimize this risk by using secure data methods as described previously.

**5.6.2 Potential study benefits**

No benefits are expected to accrue to participants in the study. However, better understanding the effect of opt-out design on clinical trial participation has the potential to increase clinical trial participation in the future, increasing the representativeness and reducing the cost of future clinical trials.

**5.6.3 Risk/benefit assessment**

Anticipated risks of this study should be minimal and the risk/benefit ratio is very favorable. We have previously outlined the procedures that will be used to prevent a breach of participant data.

1. **References**

1. Califf RM, Harrington RA. American industry and the U.S. Cardiovascular Clinical Research Enterprise an appropriate analogy? Journal of the American College of Cardiology. 2011 Aug 9;58(7):677–80.

2. Fanaroff AC, Vora AN, Chen AY, Mathews R, Udell JA, Roe MT, et al. Hospital participation in clinical trials for patients with acute myocardial infarction: Results from the National Cardiovascular Data Registry. Am Heart J. 2019 Aug;214:184–93.

3. Loewenstein G, Brennan T, Volpp KG. Asymmetric paternalism to improve health behaviors. JAMA. 2007 Nov 28;298(20):2415–7.

4. Yudin MH, Moravac C, Shah RR. Influence of an “opt-out” test strategy and patient factors on human immunodeficiency virus screening in pregnancy. Obstet Gynecol. 2007 Jul;110(1):81–6.

5. Chapman GB, Li M, Colby H, Yoon H. Opting in vs opting out of influenza vaccination. JAMA. 2010 Jul 7;304(1):43–4.

6. Mehta SJ, Khan T, Guerra C, Reitz C, McAuliffe T, Volpp KG, et al. A Randomized Controlled Trial of Opt-in Versus Opt-Out Colorectal Cancer Screening Outreach. Am J Gastroenterol. 2018;113(12):1848–54.

7. Mehta SJ, Troxel AB, Marcus N, Jameson C, Taylor D, Asch DA, et al. Participation Rates With Opt-out Enrollment in a Remote Monitoring Intervention for Patients With Myocardial Infarction. JAMA Cardiol. 2016 01;1(7):847–8.

8. Aysola J, Tahirovic E, Troxel AB, Asch DA, Gangemi K, Hodlofski AT, et al. A Randomized Controlled Trial of Opt-In Versus Opt-Out Enrollment Into a Diabetes Behavioral Intervention. Am J Health Promot. 2018;32(3):745–52.

**Supplemental Methods 2:** Email template for opt-in and opt-out outreach

**Opt-in Outreach**

Dear XXX,

Penn Medicine is offering a new program called **GAME PAD** to test ways to help patients with leg discomfort when walking increase physical activity. It is a 6 month voluntary research study and all participants receive **up to $75 and a Fitbit Inspire,** which is a watch worn on the wrist to track physical activity. Participants who are eligible to enroll will receive the Fitbit for use during their time in the study. **The program is entirely virtual and will not require you to leave your home or visit any medical facility.**

To learn more, please visit the secure website: **XXX.org.**

On the website, you can create an account and complete a few short surveys to see if the program is a good fit for you. To participate, you must be willing to wear the Fitbit watch during the day and night for 6 months. You have been selected to receive an invitation to this program. Please do not share this email or link with others.

Our team is available to answer any questions or help you get started. You can contact us by email at XXX@pennmedicine.upenn.edu or call us at XXX-XXX-XXXX. You may also reach out to us if you do not wish to be contacted about this in the future.

Thank you for taking time to consider this Program.

Sincerely,

Alexander Fanaroff, MD

Principal Investigator, GAME PAD

Assistant Professor, Penn Medicine
Email: XXX@pennmedicine.upenn.edu
Phone: XXX-XXX-XXXX

**Opt-out Outreach**

Dear XXX,

Penn Medicine is offering a new program called **GAME PAD** to test ways to help increase physical activity among patients with leg discomfort when walking. It is a 6 month voluntary research study and all participants receive **up to $75 and a Fitbit Inspire,** which is a watch worn on the wrist to track physical activity. Participants who are eligible to enroll will receive the Fitbit for use during their time in the study. You have been selected to participate in this program. If you do not wish to participate, please contact the study team. Otherwise, a member of the study team will contact you within the next week to tell you more about the program and help you enroll. **The program is entirely virtual and will not require you to leave your home or visit any medical facility.**

To learn more, please visit the secure website: **XXX.org.**

On the website, you can create an account and complete a few short surveys to see if the program is a good fit for you. To participate, you must be willing to wear the Fitbit watch during the day and night for 6 months. You have been selected to receive an invitation to this Program. Please do not share this email or link with others.

Our team is available to answer any questions or help you get started. You can contact us by email at XXX@pennmedicine.upenn.edu or call us at XXX-XXX-XXXX. You may also reach out to us if you do not wish to be contacted about this in the future.

Thank you for taking time to consider this program.

Sincerely,

Alexander Fanaroff, MD

Principal Investigator, GAME PAD

Assistant Professor, Penn Medicine
Email: XXX@pennmedicine.upenn.edu
Phone: XXX-XXX-XXXX

**Supplemental Table:** Demographics and medical history of potential participants by framing of outreach.

|  | **Opt-in**  **(n = 3909)** | **Opt-out**  **(n = 1267)** |
| --- | --- | --- |
| Age, y | 69.6 (12.3) | 69.7 (12) |
| Female | 1589 (40.6%) | 523 (41.3%) |
| White | 2622 (67.1%) | 882 (69.6%) |
| Black | 916 (23.4%) | 289 (22.8%) |
| Asian | 62 (1.6%) | 15 (1.2%) |
| Hispanic | 94 (2.4%) | 22 (1.7%) |
| Other | 215 (5.5%) | 59 (4.7%) |
| BMI, kg/m² | 29.1 (6.5) | 29 (6.5) |
| History of myocardial infarction | 751 (19.2%) | 204 (16.1%) |
| History of congestive heart failure | 1353 (34.6%) | 430 (33.9%) |
| History of stroke or cerebrovascular event | 1421 (36.4%) | 496 (39.1%) |
| History of dementia | 101 (2.6%) | 46 (3.6%) |
| History of pulmonary disease | 1408 (36%) | 425 (33.5%) |
| History of Diabetes without complications | 1620 (41.4%) | 508 (40.1%) |
| History of Diabetes with complications | 818 (20.9%) | 248 (19.6%) |
| History of renal disease | 1139 (29.1%) | 367 (29%) |

Numerical variables are presented as mean (standard deviation) while categorical variables are presented as numbers (percents).
